# Vaccination with (1–11)E2 in alum efficiently induces an antibody response to β-amyloid without affecting brain β-amyloid load and microglia activation in 3xTg mice

**DOI:** 10.1007/s40520-019-01414-0

**Published:** 2019-11-22

**Authors:** Francesca Mantile, Angelo Capasso, Nadia Villacampa, Maria Donnini, Giovanna L. Liguori, Gabriela Constantin, Piergiuseppe De Berardinis, Michael T. Heneka, Antonella Prisco

**Affiliations:** 1 Istituto di Genetica e Biofisica Adriano Buzzati Traverso (IGB), CNR, Naples, Italy; 2 Deutsches Zentrum fur Neurodegenerative Erkrankungen (DZNE), Bonn, Germany; 3 Università degli Studi di Verona Dipartimento di Medicina Sezione di Patologia Generale, Verona, Italy; 4 Istituto di Biochimica e Biologia Cellulare (IBBC), CNR, Naples, Italy

**Keywords:** Alzheimer’s disease, Immunization, β-amyloid, Antibody response

## Abstract

Immunization against β-amyloid (Aβ) is pursued as a possible strategy for the prevention of Alzheimer’s disease (AD). In clinical trials, Aβ 1–42 proved poorly immunogenic and caused severe adverse effects; therefore, safer and more immunogenic candidate vaccines are needed. Multimeric protein (1–11)E2 is able to induce an antibody response to Aβ, immunological memory, and IL-4 production, with no concomitant anti-Aβ T cell response. Antisera recognize Aβ oligomers, protofibrils, and fibrils. In this study, we evaluated the effect of prophylactic immunization with three doses of (1–11)E2 in alum in the 3xTg mouse model of AD. Immunization with (1–11)E2 efficiently induced anti-Aβ antibodies, but afforded no protection against Aβ accumulation and neuroinflammation. The identification of the features of the anti-Aβ immune response that correlate with the ability to prevent Aβ accumulation remains an open problem that deserves further investigation.

## Introduction

Although numerous factors contribute to Alzheimer’s disease (AD) pathogenesis, the accumulation of β-amyloid (Aβ) in the brain is the most extensively validated therapeutic target [1, 2]. Among the anti-Aβ therapeutic approaches, one that has been extensively developed is immunotherapy, both active and passive; several vaccines that induce anti-Aβ antibodies and monoclonal antibodies targeting Aβ have been clinically tested [3, 4]. Immunogenicity and safety are the major roadblocks in the development of a vaccine against Aβ. In the clinical trial of AN1792, consisting of full-length Aβ42 with QS-21 adjuvant, only a minority of vaccinees (50 out of 300) produced antibodies and 6% of treated participants developed aseptic meningoencephalitis [3, 4]. The adverse reaction was attributed to the infiltration of Th1 T cells in the brain. Importantly, responders demonstrated significantly reduced functional decline compared with placebo-treated patients and post-mortem neuropathological examination demonstrated clearance of plaques. To avoid adverse reactions, most second generation vaccines direct the antibody response against regions of Aβ that do not contain T cell epitopes such as the N-terminus [3, 4].

We have previously identified as a promising candidate vaccine, (1–11)E2, a fusion protein that includes the first 11 N-terminal residues of Aβ, i.e. DAEFRHDSGYE, and bacterial protein domain E2, which self-assembles into a 60-mer complex.

(1–11)E2 is able to induce an antibody response to β-amyloid, immunological memory, and IL-4 production, with no concomitant anti-Aβ T cell response and induces antibodies that recognize β-amyloid oligomers, protofibrils, and fibrils [5–8].

In this study, we show that a three-dose vaccination with (1–11)E2 in alum did not reduce β-amyloid load in 3xTg mice, despite efficiently inducing anti-β-amyloid antibodies.

## Materials and methods

### Mice

The 3xTg-AD mice [9] were purchased from The Jackson Laboratory (Sacramento, CA).

### Immunization protocol

The (1–11)E2 protein was produced in *E. coli* [5, 7, 8]. Each vaccine dose included 130 μg of (1–11)E2 adsorbed on 5 μg Alhydrogel 2%, in a final volume of 100 μl, injected subcutaneously. Mice received three doses, at age 3, 7, and 12 months, and were killed at age 13 months. Mice were bled at day 14, 90, 148, 240, and 300 from the first immunization. The anti-Aβ antibody titer was measured by ELISA, as described [5].

### Histology

Tissue was prepared as previously described [10]. Right hemispheres were paraffin embedded and stored at +4 °C. After deparaffinization and antigen retrieval in 10 mM sodium citrate buffer, pH 6.0, sections. (10 μm) were stained as described [10] with rabbit anti-Iba1 (1:1000, 019-19741,Wako) and mouse anti-Aβ Mab 6E10 (1:500, SIG-39320-500, Covance).

For thioflavin S staining, after deparaffinization and antigen retrieval in 10 mM sodium citrate buffer, pH 6.0, slices were rinsed in water, incubated in 0.01% thioflavin S in water, and differentiated in 70% ethanol.

### Aβ measures in brain extracts

Brain protein extraction was performed as described [10]. Quantitative determination of amyloid-β_1–40_ and amyloid-β_1–42_ in RIPA, SDS, and FA fractions was performed by electrochemiluminescence ELISA (Meso Scale Discovery) [10].

### Statistical analysis

The significance of differences was evaluated with the unpaired *t* test.

## Results

### Immunization with (1–11)E2 in alum induced a persistent anti-β-amyloid antibody response in 3xTg mice

To test the preventative efficacy of (1–11)E2 vaccination on β-amyloid accumulation, we vaccinated 3xTg mice (*n* = 10) with three doses of (1–11)E2 in alum, given at age 3, 7, and 12 months; a control group (*n* = 10) received PBS injections.

**Fig. 1:**
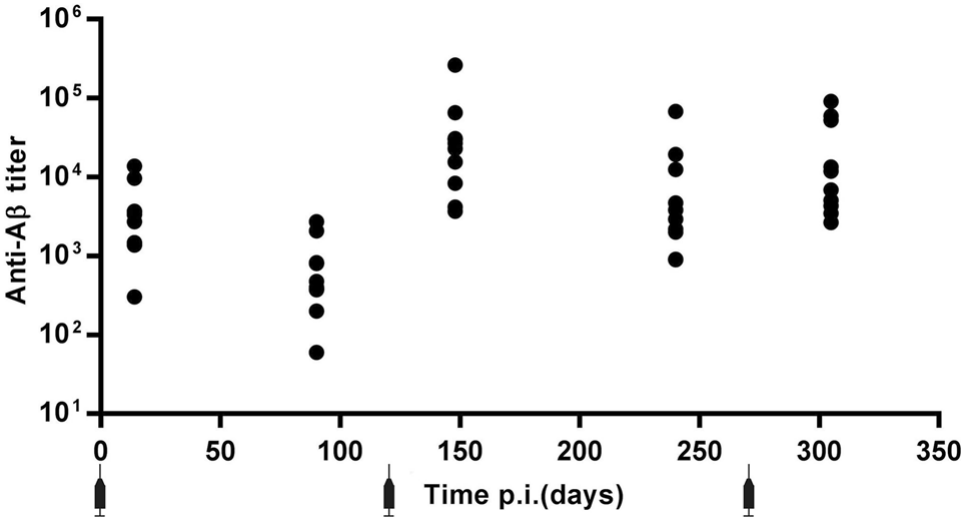
(1–11)E2 efficiently induced anti-β-amyloid responses. The dot plot shows the anti-Aβ IgG titer at days 14, 90, 148, 240, and 300 after the first immunization with (1–11)E2 in 3xTg mice. Each dot represents one mouse

We monitored the anti-Aβ antibody titers over time (Fig. 1). All mice immunized with (1–11)E2 displayed a measurable anti-Aβ antibody titer 2 weeks after the first injection (range 1: 300 to 1:13,000, geometric mean titer GMT 1:2590). Titers declined over time but were still measurable in all mice 3 months after the first dose. When mice were killed at age 13 months, their anti-Aβ antibody titer ranged from 1:2700 to 1:90,000, and the GMT was 1:12,000 (Fig. 1).

Thus, the immunization of 3xTg mice with three doses of (1–11)E2 in alum induced a persistent anti-β-amyloid antibody response in all individuals.

### Prophylactic (1–11)E2 immunization did not reduce β-amyloid load and neuroinflammation

**Fig. 2:**
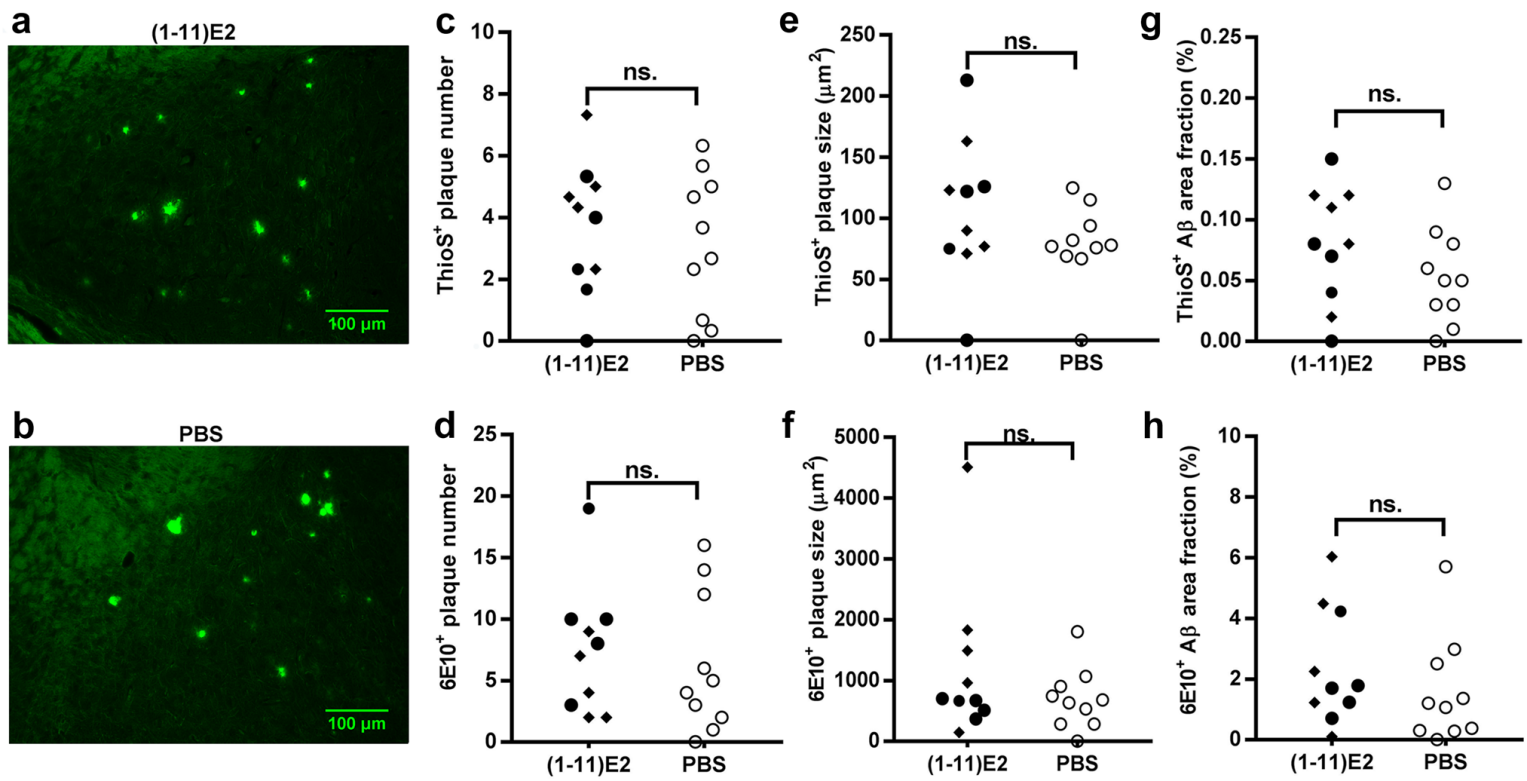
(1–11)E2 vaccination did not reduce Aβ plaques number and size and the area fraction occupied by plaques. Representative thioflavin S histochemistry in the hippocampus of 13-month-old 3xTg mice immunized with (1–11)E2 (**a**) or PBS (**b**). Dot plots show the number (**c**), plaque size (**e**), and area fraction (**g**) of thioflavin S^+^ amyloid plaques in 3xTg mice immunized with (1-11)E2 (black symbols) or PBS (white symbols). Each dot represents one mouse. Quantification of 6E10^+^ amyloid plaques number (**d**), plaque size, (**f**) and Aβ area fraction (**h**) in 3xTg mice (*n* = 10) immunized with (1–11)E2 (black) or PBS (white). Each dot represents one mouse. In the (1–11)E2 group, diamonds indicate high responders (anti-Aβ titer at day 300 above 1:10,000) and circles indicate low responders (titer below 1:10,000). No statistically significant difference was observed between (1–11)E2 vaccinated mice and PBS controls

**Fig. 3:**
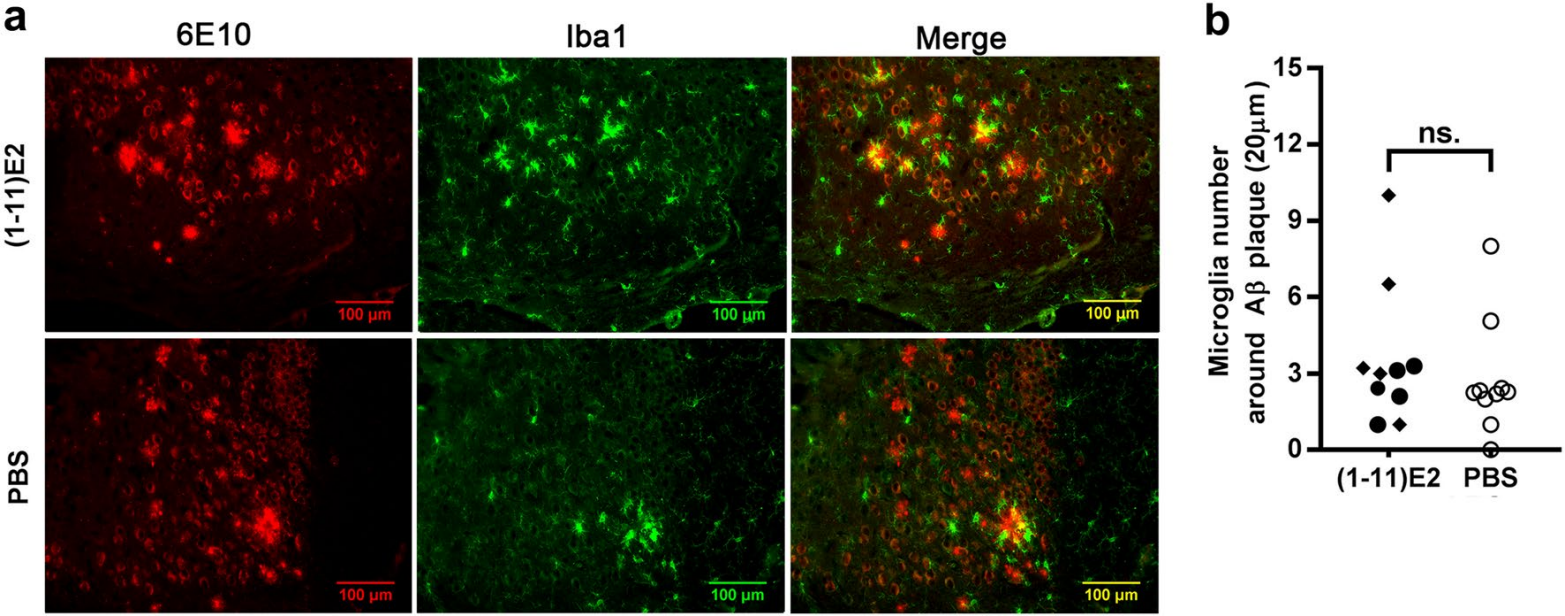
(1–11)E2 vaccination does not reduce the number of microglial cells around Aβ plaques. **a** Representative brain section of the hippocampus of 13-month-old 3xTg mice immunized with (1–11)E2 or PBS analyzed by immunohistochemistry for amyloid plaques (6E10) and microglia (Iba1). **b** The dot plot represents the number of Iba1 + microglia cells within a distance of 20 μm from β-amyloid plaques. Each dot represents one mouse, diamonds and circles represent high and low anti-Aβ responders, as in Fig. 2. No statistically significant difference was observed between (1–11)E2 vaccinated mice and PBS controls

**Fig. 4:**
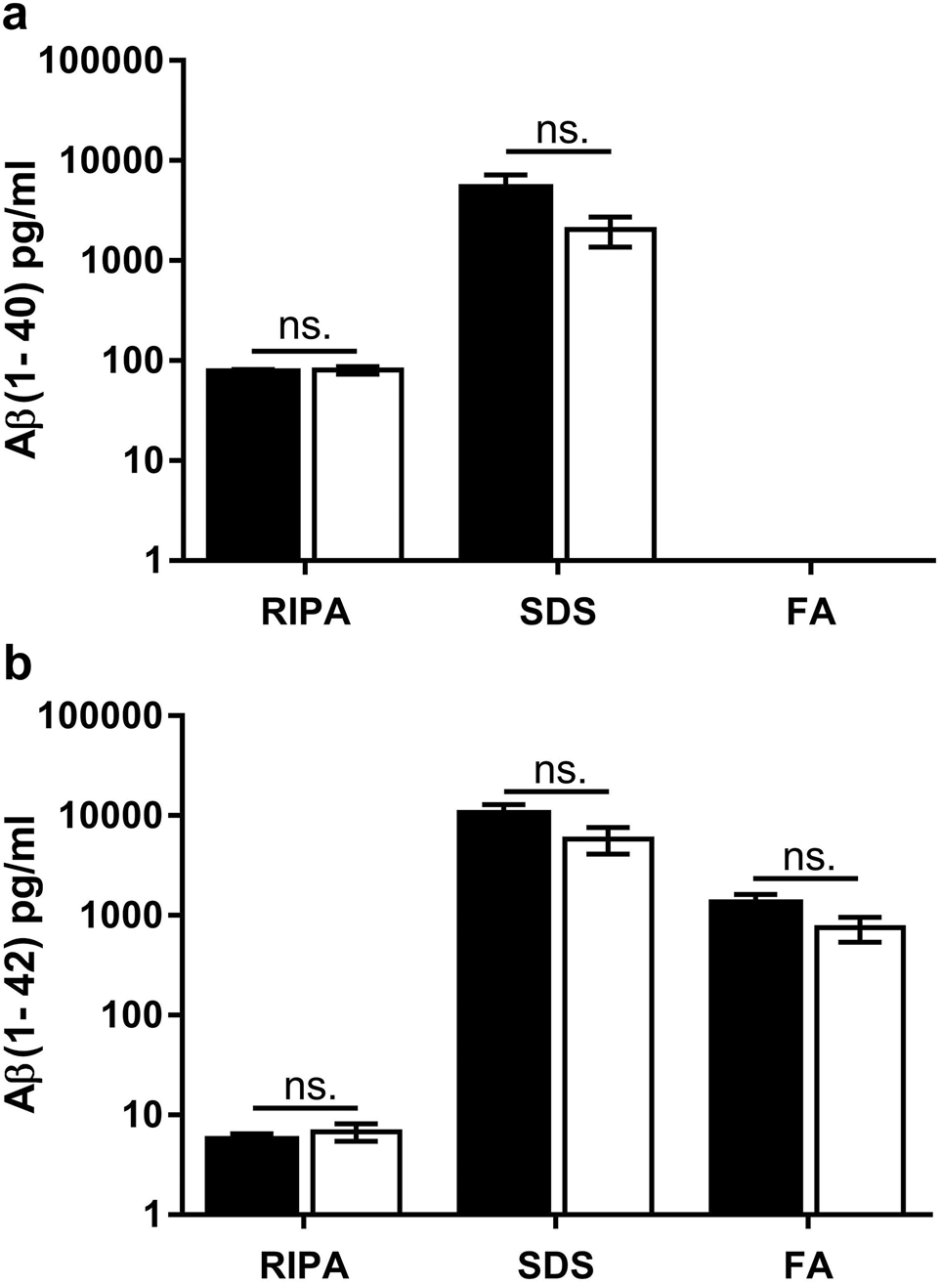
(1–11)E2 vaccination does not reduce soluble and insoluble Aβ in the brain. Quantification by ELISA of amyloid-β_1–40_ (**a**) and amyloid-β_1–42_ (**b**) concentration in RIPA, SDS, and FA fractions from 13-month-old 3xTg mice (*n* = 10) immunized with (1–11)E2 (black column) or PBS (white column). Error bars represents SEM. No statistically significant difference was observed between (1–11)E2 vaccinated mice and PBS controls

We next analyzed the β-amyloid load in the brain of vaccinated mice and controls, in 13-month-old mice. For each brain, the right hemisphere was analyzed histochemically (Figs. 2, 3) and the left hemisphere was used to generate protein extracts that were analyzed by ELISA for their Aβ_1–40_ and Aβ_1–42_ content (Fig. 4).

Thioflavin S^+^ plaques did not differ in number and size between (1–11)E2 vaccinated mice and PBS controls (Fig. 2). Only 0–14 thioflavin S^+^ plaques were observed in each section, all located in the hippocampus and in particular in the subiculum (Fig. 2). The plaques recognized by the 6E10 anti-β-amyloid antibody, which also recognizes diffuse deposits that are not stained by thioflavin S, did not differ in number and size between (1–11)E2 vaccinated mice and PBS controls (Fig. 2). The number of microglial cells surrounding plaques was not affected by the (1–11)E2 vaccination (Fig. 3). The amount of Aβ_1–40_ and Aβ_1–42_ in protein extracts did not differ between (1–11)E2 vaccinated mice and PBS controls (Fig. 4).

Thus, we conclude that (1–11)E2 immunization failed to reduce β-amyloid load and neuroinflammation despite inducing anti-Aβ antibodies.

## Discussion

In this study, we observed that the multimeric protein vaccine (1–11)E2 is immunogenic in 3xTg mice, triggering the production of anti-Aβ antibodies in all individuals. However, we observed no effect of immunization on brain amyloid burden and microglia at age 13 months.

In our studies, the immunization was performed at 12–15 weeks of age, well before the formation of β-amyloid plaques. We observed no difference in β-amyloid load between high responders, with antibody titers above 1:10,000 and low responders, with titers below 1:10,000. Thus, it seems unlikely that in this experiment, the failure of (1–11)E2 to interfere with Aβ accumulation was due to an insufficient titer or inappropriate timing of the response (“too little, too late”). In a previous study in APP PSEN1 mice immunized with a phage-based vaccine, a treatment group with a titer above 1:10,000 afforded reduced β-amyloid load [11–13] leading us to hypothesize that a threshold anti-Aβ antibody titer is required for efficacy. One drawback of the immunization schedule utilized here, which included intervals of several months between doses to favor immunological memory [8, 14, 15], is that it led to comparatively low anti-Aβ titers when mice were around age 5 months old.

It is not possible to establish from this study whether the failure to reduce β-amyloid was due to the fine specificity, affinity, or isotype of the antibodies induced by (1–11)E2. The identification of the features of the immune response that correlate with the ability to interfere with the accumulation of β-amyloid remains an open problem that deserves further investigation. It is essential to compare different vaccination protocols, in terms of quantitative and qualitative features of the immune response, to identify correlates of efficacy.
